# Anthropometric orofacial measures of newborns and their relationship with sex, weight and height

**DOI:** 10.1590/2317-1782/20212020114

**Published:** 2022-01-05

**Authors:** Silvia Márcia Andrade Campanha, Roberta Lopes de Castro Martinelli, Durval Batista Palhares

**Affiliations:** 1 Programa de Pós-graduação em Saúde e Desenvolvimento na Região Centro-Oeste, Faculdade de Medicina – FAMED, Universidade Federal de Mato Grosso do Sul – UFMS - Campo Grande (MS), Brasil.; 2 Hospital Santa Therezinha - Brotas (SP), Brasil.

**Keywords:** Face, Newborns, Anthropometry, Birth Weight, Body Height, Body Weights and Measures

## Abstract

**Purpose:**

To compare orofacial anthropometric measurements, with weight, height and sex of newborns.

**Methods:**

Observational cross-sectional study carried out with 130 newborns on exclusive breastfeeding. Data collection was performed by properly trained and calibrated speech therapists. The orofacial measurements of the newborns were performed with flexible and transparent ruler 10 cm long, in the following segments: heights of the upper third(tr-g), the middle third(g-sn) and the lower third on the face (sn-gn); filter height (sn-Is), distance between the corner of the eye and the labial commissure on the right and left side (ex-ch). Weight and height measurements were collected from the newborns' medical records. The data were submitted to statistical analysis, using the Mann-Whitney test, adopting a significance level of 5%.

**Results:**

Of the 130 newborns, 61 were male and 69 female. The median weight was 3.3 kg and the median height was 49 cm. There was significant difference between weight and measurement distance between the corner of the eye and the left and right labial commissure (ex-ch). There was no significant difference in orofacial measurements with sex and height.

**Conclusion:**

There was no difference in orofacial anthropometric measurements of full-term newborns when compared with sex and height; however, when compared to weight, there is a difference in the measurements of the distance between the corner of the eye and the labial commissure on the right and left side.

## INTRODUCTION

The dimensions of the human body are affected by anatomophysiological and ethnic-racial factors, as well as by sex and age^(1)^. The human face has its own characteristics according to the configuration of the craniofacial structures, being one of the regions that presents more variations according to race^(2)^.

Anthropometry, a word derived from the Greek *anthropos* (man) and *metrom* (measure), is the science that studies the measurements and proportions of the human body^(3)^, and can provide objective data on craniofacial morphology through a set of head and face measurements^(4)^, using simple, low-cost, non-invasive and risk-free techniques^(5-7)^. Thus, the anthropometric orofacial assessment is based on locating specific points on the face and performing measurements with a caliper or measuring tape^(2-9)^, providing reference data of normality for a wide variety of orofacial measurements^(6)^. As disadvantages, the literature refers to errors related to the reading of the instruments used, as well as inadequate training or improper use of the instruments by the evaluator^(3)^.

The reliability of anthropometric orofacial measurements depends on the exact location of the anthropometric points and the maintenance of the proper head position^(3,6)^, being very useful in the diagnosis of craniofacial anomalies^(6)^.

In the consulted databases, references of normality of orofacial anthropometric measurements for children^(2,5-7 ^,)(^ 10-14)^, adolescents^(15)^ and adults^(16,17)^ were found. However, there is a scarcity of studies referring to the measurements of the face of Brazilian newborns^(18)^.

The knowledge of these measurements is very important for the development of assessment instruments, with the objective of detecting abnormalities present in various alterations that can impair the correct development of the orofacial functions.

Thus, the aim of this study was to compare the orofacial anthropometric measurements with weight, height and sex of newborns that were born at Hospital Universitário Maria Aparecida Pedrossian of Universidade Federal de Mato Grosso do Sul.

## METHODS

This was an observational cross-sectional study that was approved by the Research Ethics Committee of Universidade Federal de Mato Grosso do Sul under number 1,514,715. All parents or guardians were informed about the objectives and procedures of the study and signed the Informed Consent Form.

A total of 130 brown and white newborns, aged from one to five days old, from the Rooming-in Sector of the University Hospital of Universidade Federal de Mato Grosso do Sul, from June to December 2016, were evaluated.

This study included full-term newborns, with APGAR greater than or equal to eight, who were exclusively breastfed. Excluded from this study: Newborns from the indigenous and quilombola population; preterms; the ones with perinatal complications, unstable clinical conditions, presence of craniofacial anomalies, neurological diseases, genetic syndromes visible at the time of the evaluation, artificial feeding, as well as the newborns of postpartum women with positive serum for the Human Immunodeficiency Virus (HIV).

Assessments were performed 24 hours after birth. Data collection was performed by four evaluators, the researcher and three speech-language pathologists from the team of the University Hospital of Universidade Federal de Mato Grosso do Sul, who were duly trained and calibrated. For this phase, a pilot study was carried out with the participation of 14 newborns. At the end of the training, the evaluators reached a degree of agreement above 90%. In the pilot study, a hardened stainless steel digital caliper (brand Profield- Electronic Digital Caliper, 150 mm, resolution: 0.01mm/0.0005”, precision:+or-0.02mm/+or-0.001”, repeatability of 0.01mm/0.0005”, made in Germany), the measuring tape and the flexible 10-cm ruler were used for the orofacial measurements.

**Figure 1:**
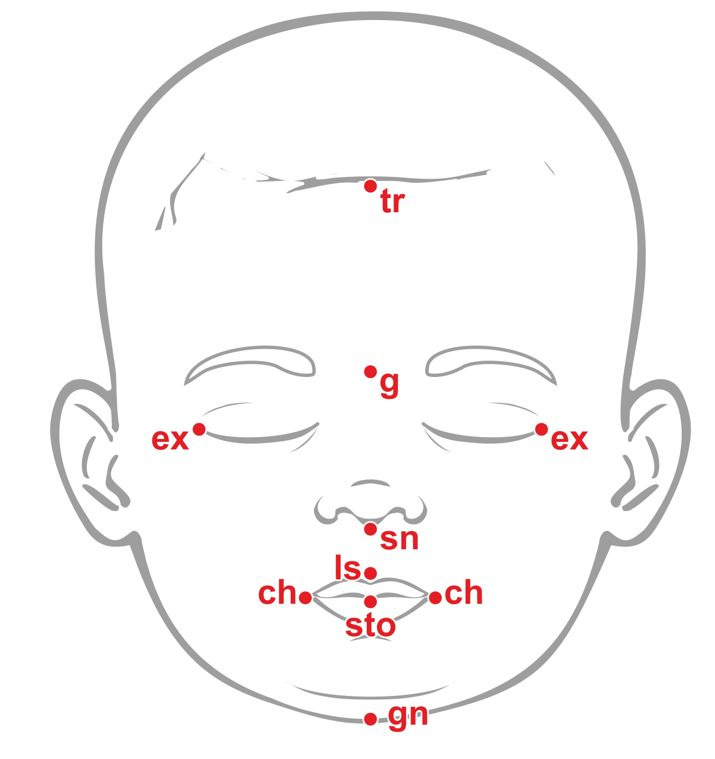
Antropometric points used for orofacial measurementes of newborns

Figure 1 shows the points that served as reference for the orofacial anthropometric measurements: trichion (tr), point located at the hairline insertion in the midline of the forehead; glabella (g), most prominent point on the midline between the eyebrows; subnasale (sn), point located medially at the union of the lower border of the nasal septum with the surface of the upper lip; labiale superius (ls), located medially on the vermilion line of the upper lip; stomion (sto), imaginary point located in the medial region of the intersection between the median vertical line of the face and the horizontal line of the rim of the mouth, when the lips are closed and the teeth occluded; gnathion (gn), point located in the lowest region of the lower border of the mandible; outer corner or excanthion of the eye (ex), medially located on the outer border of the eye, taking the hard tissue as a reference; cheilion (ch), point located in the labial commissure^(2,6,7,12)^.

Caption: tr = trichion; g = glabella; ex = outer corner (or excanthion) of the eye; sn = subnasale; ls = labiale superius; ch = cheilion; sto = stomion; gn = gnathion

There was no difference in values ​​between the caliper, the measuring tape and the transparent ruler in the first 14 newborns evaluated. So, the transparent malleable ruler was chosen for the evaluation, as it is considered safer for newborns as it is not sharp as a caliper is. Because the malleable ruler is transparent, it was possible to mark the corresponding value with a felt-tip pen at the time of evaluation. The statistical analysis of these measures was not performed in the pilot study. They were only compared between the evaluators, with the different instruments.

**Figure 2:**
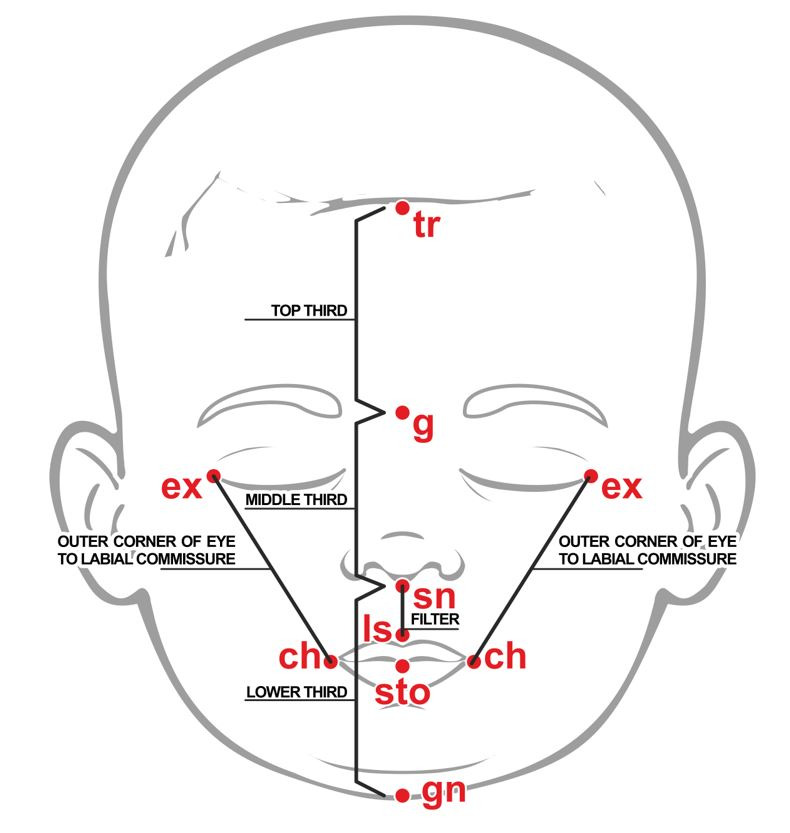
Anthropometric orofacial measurements of the newborns

After verifying the feasibility of obtaining orofacial measurements in the pilot study, 130 newborns were evaluated. The newborns' orofacial measurements were performed with a flexible and transparent 10cm-long ruler, in the following segments: heights of the upper third (tr-g), the middle third (g-sn) and the lower third of the face (sn-gn); filter height (sn-Is); and distance between the corner of the eye and the labial commissure on the right and left side (ex-ch), as shown in Figure 2. The measurements were initially taken in centimeters (cm), converted to mm and each one was performed three times. The arithmetic mean of each measure was subsequently calculated. The measurements were taken with the baby sleeping, lying in a hospital crib, in the supine position, with the lips closed, right after the feed. The evaluators wore gloves throughout the evaluation and cleaned the ruler with hydrated ethyl alcohol 70º INPM (Instituto Nacional de Pesos e Medidas; National Institute of Weights and Measures), before and after the procedure.

After the measurements, information regarding the sex, weight and height of the newborns was collected from the newborns' medical records. To compare weight and height with orofacial anthropometric measurements, values that were lower than or equal to the median and greater than the median of these variables were used.

The data obtained were tabulated and submitted to statistical analysis. The sample was characterized by descriptive data analysis: mean, standard deviation, coefficient of variation (CV), minimum (Min) and maximum (Max) values, with a 95% confidence interval. The IBM SPSS Statistics program (Statistical Package for the Social Sciences), in its version 25.0, was used to obtain the results, and the Mann-Whitney test was applied considering a significance level of 5%.

## RESULTS

Of the 130 newborns evaluated, 69 (53%) were female and 61 (47%) were male. The average number of days of life of the newborns was 1.68 days. The median weight was 3.3 kg and the median height was 49 cm.

Table 1 presents the results obtained by comparing the anthropometric orofacial measurements with the sex of the newborns, showing that no significant differences were found between the orofacial measurements of the newborns regarding the sex.

**Table 1:** Comparison of anthropometric **orofacial** measurements with sex

Variable	Sex	n	Mean	SD	Minimum	Maximum	Percentile 25	Percentile 50 (Median)	Percentile 75	p Value
Upper third of the face **(mm**)	F	69	32.5	3.10	26.0	40.0	30.0	32.0	35.0	0.417
	M	61	32.7	3.00	25.0	40.0	30.0	33.0	35.0	
	Total	130	32.6	3.10	25.0	40.0	30.0	33.0	35.0	
Middle third of the face (**mm)**	F	69	29.6	2.30	25.0	38.0	28.0	30.0	30.0	0.883
	M	61	29.5	2.20	23.0	35.0	28.0	30.0	30.0	
	Total	130	29.6	2.30	23.0	38.0	28.0	30.0	30.0	
Lower third of the face (**mm)**	F	69	29.3	3.00	22.0	40.0	27.0	30.0	30.0	0.070
	M	61	28.4	3.50	21.0	40.0	26.0	28.0	30.0	
	Total	130	28.9	3.20	21.0	40.0	27.0	29.0	30.0	
Outer corner of eye to labial commissure LS **(mm)**	F	69	39.4	2.30	33.0	44.0	38.0	40.0	40.0	0.814
	M	61	39.1	2.70	32.0	45.0	38.0	40.0	40.0	
	Total	130	39.3	2.50	32.0	45.0	38.0	40.0	40.0	
Outer corner of eye to labial commissure RS (**mm)**	F	69	38.7	2.80	25.0	44.0	38.0	39.0	40.0	0.651
	M	61	38.9	2.80	32.0	45.0	38.0	40.0	40.0	
	Total	130	38.8	2.70	25.0	45.0	38.0	39.5	40.0	
Filter (**mm**)	F	69	8.70	1.30	5.00	11.0	8.00	9.00	10.0	0.944
	M	61	8.80	1.20	5.00	11.0	8.00	9.00	10.0	
	Total	130	8.70	1.30	5.00	11.0	8.00	9.00	10.0	

Mann-Whitney Test

Caption: F = Female; M = Male; LS = Left Side; RS = Right Side; SD = Standard Deviation

When comparing the anthropometric orofacial measurements with the medians of weight, there was a significant difference between the weight and the distance between the outer corner of the eye and the right and left labial commissure (ex-ch), as described in Table 2.

**Table 2:** Comparison of anthropometric **orofacial** measurements with weight

Variable	Weight	n	Mean	SD	Minimum	Maximum	Percentile 25	Percentile 50 Value/Median	Percentile 75	P Value
Upper third of the face (**mm)**	≤ the median	65	32.6	3.10	25.0	40.0	30.0	33.0	35.0	0.911
	> the median	65	32.6	3.10	26.0	40.0	30.0	32.0	35.0	
	Total	130	32.6	3.10	25.0	40.0	30.0	33.0	35.0	
Middle third of the face **(mm)**	≤ the median	65	29.5	2.20	25.0	35.0	28.0	30.0	30.0	0.931
	> the median	65	29.6	2.30	23.0	38.0	28.0	30.0	30.0	
	Total	130	29.6	2.30	23.0	38.0	28.0	30.0	30.0	
Lower third of the face (**mm**)	≤ the median	65	28.9	3.30	21.0	40.0	27.0	29.0	30.0	0.756
	> the median	65	28.8	3.20	22.0	40.0	26.5	29.0	30.0	
	Total	130	28.9	3.20	21.0	40.0	27.0	29.0	30.0	
**OCELC** LS **(mm)**	≤l the median	65	38.7	2.70	32.0	45.0	37.0	39.0	40.0	0.003^*^
	**>** the median	65	39.9	2.00	35.0	44.0	39.0	40.0	41.0	
	Total	130	39.3	2.50	32.0	45.0	38.0	40.0	40.0	
**OCELC** RS **(mm**)	≤l the median	65	38.3	3.10	25.0	45.0	37.0	39.0	40.0	0.016*
	**>** the median	65	39.2	2.20	33.0	44.0	38.0	40.0	40.0	
	Total	130	38.8	2.70	25.0	45.0	38.0	39.5	40.0	
Filter **(mm)**	≤l the median	65	8.60	1.20	5.00	11.0	8.00	9.00	9.00	0.108
	> the median	65	8.90	1.30	5.00	11.0	8.00	9.00	10.0	
	Total	130	8.70	1.30	5.00	11.0	8.00	9.00	10.0	

Mann-Whitney Test

* Statistical Significance

Caption: OCELC = Outer corner of eye to labial commissure; LS = Left Side; RS = Right Side; SD = Standard Deviation

The results obtained by comparing the anthropometric orofacial measurements and the height medians are presented in Table 3, showing that no significant differences were observed between the newborns' orofacial measurements regarding the height.

**Table 3:** Comparison of anthropometric orofacial measurements with height

Variable	Height	n	Mean	SD	Minimum	Maximum	Percentile 25	Percentile 50 (Median)	Percentile 75	P Value
Upper third of the face **(mm)**	≤l the median	66	32.4	2.90	25.0	40.0	30.0	33.0	35.0	0.820
	> the median	64	32.8	3.30	26.0	40.0	30.0	32.0	35.0	
	Total	130	32.6	3.10	25.0	40.0	30.0	32.0	35.0	
Middle third of the face **(mm**)	≤l the median	66	29.2	2.20	23.0	35.0	28.0	30.0	30.0	0.057
	> the median	64	29.9	2.30	25.0	38.0	28.0	30.0	31.0	
	Total	130	29.6	2.30	23.0	38.0	28.0	30.0	30.0	
Lower third of the face **(mm)**	≤l the median	66	28.6	3.50	21.0	40.0	26.0	28.0	30.0	0.280
	> the median	64	29.1	2.90	24.0	40.0	27.0	29.0	30.8	
	Total	130	28.9	3.20	21.0	40.0	27.0	29.0	30.0	
**OCELC** LS **(mm)**	≤l the median	66	39.0	2.70	32.0	45.0	38.0	40.0	40.0	0.095
	> the median	64	39.6	2.20	32.0	44.0	38.0	40.0	41.0	
	Total	130	39.3	2.50	32.0	45.0	38.0	40.0	40.0	
**OCELC** RS (**mm**)	≤l the median	66	38.7	3.10	25.0	45.0	37.8	39.0	40.0	0.633
	> the median	64	38.9	2.30	33.0	44.0	38.0	40.0	40.0	
	Total	130	38.8	2.70	25.0	45.0	38.0	39.5	40.0	
Filter (**mm**)	≤l the median	66	8.60	1.30	5.00	10.0	8.00	9.00	9.30	0.438
	> the median	64	8.80	1.30	5.00	11.0	8.00	9.00	10.0	
	Total	130	8.70	1.30	5.00	11.0	8.00	9.00	10.0	

Mann-Whitney Test

Caption: OCELC = Outer corner of eye to labial commissure; LS = Left Side; RS = Right Side; SD = Standard Deviation

## DISCUSSION

The present study was motivated by the scarcity of data on anthropometric orofacial measurements of newborns, being a pioneer in the investigation of these measurements in the Midwest region of Brazil.

The mean weight of the newborns evaluated was 3,302 g and the mean height was 49 cm, values ​close to those found by Oliveira et al.^(19)^ in 450 newborns evaluated, whose mean weight was 3,278 g and mean height 48.8 cm.

When comparing anthropometric orofacial measurements regarding the sex of the newborns, there was no statistical difference, showing that sex does not seem to interfere in these variables. These findings differ from the results found by Medeiros et al.^(18)^, who reported that there are differences in orofacial measurements between the sexes right at birth, which are always greater in males. In other studies, significant differences were found between filter height and sex^(20,21)^, and distance of the mouth commissures between sexes^(4)^.

Differences in the results of these surveys were identified, which can be explained by the fact that these studies used different methodologies and were carried out in different regions of several countries. Literature reports that head and face dimensions vary according to race and geographic area^(4)^.

This study also showed a statistically significant difference when comparing the anthropometric orofacial measurements of the distance between the corner of the eye and the right and left labial commissure with birth weight. Thus, heavier weights allow us to observe effectively greater values ​​of the distance between the corner of the eye and the left and right labial commissure; and, as for lighter weights, to observe smaller values. The scarcity of studies does not allow comparing the data found. However, it is possible to infer that the measurements of the distance between the corner of the eye and the labial commissure are influenced by the weight. An explanation for this finding may lie in the fact that in babies with heavier weights the fat bags are possibly more bulky, and as the malleable ruler molds itself to the structure, the measure of the distance between the corner of the eye and the labial commissure may increase.

When comparing height with the anthropometric orofacial measurements, no statistically significant differences were found. The scarce literature in this area and for this population made it difficult to compare the findings with other studies. This is probably because facial growth is more accentuated in the first two years of life, through breastfeeding, and not necessarily at birth.

As a limitation of this research, we can mention the lack of pairing of the groups regarding sex, with a predominance of females. Considering the Brazilian ethnic miscegenation, the values found in the State of Mato Grosso do Sul cannot be generalized to newborns from other regions of the country. Thus, future multicenter studies, using the same methodology of the proposed study, with a larger sample, will contribute to the knowledge of the profile of the orofacial measurements of Brazilian newb orns.

## CONCLUSION

There was no difference in the orofacial anthropometric measurements of full-term newborns when compared with sex and height. However, when compared with weight, there is difference in the measurements of the distance between the corner of the eye and the labial commissure of the right and left sides.
