# Supplementary material for: Anthropometric orofacial measures of newborns and their relationship with sex, weight and height
Source: Codas. 2022 Jan 5;34(1):e20200114. doi: 10.1590/2317-1782/20212020114 (PMC9886102; doi:10.1590/2317-1782/20212020114)
Supplement: Translation

# Translation: Medidas antropométricas orofaciais de recém-nascidos e sua relação com sexo, peso e altura

**Authors:** Silvia Márcia Andrade Campanha, Roberta Lopes de Castro Martinelli, Durval Batista Palhares

**Affiliations:** 1 Programa de Pós-graduação em Saúde e Desenvolvimento na Região Centro-Oeste, Faculdade de Medicina – FAMED, Universidade Federal de Mato Grosso do Sul – UFMS - Campo Grande (MS), Brasil.; 2 Hospital Santa Therezinha - Brotas (SP), Brasil.

**Keywords:** Face, Recém-nascido, Antropometria, Peso ao Nascer, Estatura, Pesos e Medidas Corporais

## Abstract

**Objetivo:**

Comparar as medidas antropométricas orofaciais, com peso, altura e sexo de recém-nascidos.

**Método:**

Estudo transversal observacional realizado com 130 recém-nascidos em aleitamento materno exclusivo. A coleta de dados foi realizada por fonoaudiólogas devidamente treinadas e calibradas. As medidas orofaciais dos recém-nascidos foram realizadas com uma régua maleável e transparente de 10 cm de comprimento, nos seguintes segmentos: alturas do terço superior (tr-g), do terço médio (g-sn) e do terço inferior da face (sn-gn); altura do filtro (sn-Is); distância entre o canto do olho e a comissura labial do lado direito e esquerdo (ex-ch). A medida do peso e da altura foram coletadas nos prontuários dos recém-nascidos. Os dados foram submetidos à análise estatística, sendo aplicado o teste de Mann-Whitney, adotando nível de significância de 5%.

**Resultados:**

Dos 130 recém-nascidos, 61 eram do sexo masculino e 69 do sexo feminino. A mediana do peso foi de 3,3 kg e da altura de 49 cm. Houve diferença significativa entre o peso e a medida da distância entre o canto do olho e a comissura labial do lado direito e esquerdo (ex-ch). Não foi encontrada diferença significativa das medidas orofaciais com sexo e altura.

**Conclusão:**

Não houve diferença nas medidas antropométricas orofaciais de recém-nascidos a termo quando comparadas com sexo e altura; porém, quando comparadas com peso, há diferença nas medidas da distância entre o canto do olho e a comissura labial do lado direito e esquerdo.

## INTRODUÇÃO

As dimensões do corpo humano são afetadas por fatores anatomofisiológicos e étnico-raciais, bem como, pelo sexo e idade^(1)^. A face humana apresenta características próprias de acordo com a configuração das estruturas craniofaciais, sendo uma das regiões que mais sofre variações de acordo com a raça^(2)^.

A antropometria, palavra derivada do grego *anthropos* (homem) e *metrom* (medida), é a ciência que estuda as medidas e proporções do corpo humano^(3)^, podendo fornecer dados objetivos da morfologia craniofacial por meio de um conjunto de medidas da cabeça e face^(4)^, utilizando técnicas simples, de baixo custo, não invasivas e sem riscos^(5-7)^. Assim, a avaliação antropométrica orofacial é baseada na localização de pontos específicos da face e execução de medições com paquímetro ou fita métrica^(2-9)^, fornecendo dados de referência da normalidade para uma grande variedade de medidas orofaciais^(6)^. Como desvantagens, a literatura refere os erros referentes à leitura dos instrumentos utilizados, bem como, o treino inadequado ou uso impróprio dos instrumentos pelo avaliador^(3)^.

A confiabilidade das medidas antropométricas orofaciais depende da localização exata dos pontos antropométricos e da manutenção da posição adequada da cabeça^(3,6)^, tendo grande utilidade no diagnóstico de anomalias craniofaciais^(6)^.

Nas bases de dados consultadas, foram encontradas referências de normalidade de medidas antropométricas orofaciais para crianças^(2,5-7 ^,)(^ 10-14)^, adolescentes^(15)^ e adultos^(16,17)^porém, há uma escassez de estudos referentes às medidas da face de recém-nascidos brasileiros^(18)^.

O conhecimento dessas medidas é de suma importância para a elaboração de instrumentos de avaliação, com o objetivo de detectar anormalidades presentes em várias alterações, que podem comprometer o correto desenvolvimento das funções orofaciais.

Assim, o objetivo deste estudo foi comparar as medidas antropométricas orofaciais, com peso, altura e sexo de recém-nascidos nascidos no Hospital Universitário Maria Aparecida Pedrossian da Universidade Federal de Mato Grosso do Sul.

## MÉTODOS

Esta foi uma pesquisa transversal observacional, tendo sido aprovada pelo Comitê de Ética em Pesquisa da Universidade Federal de Mato Grosso do Sul sob no 1.514.715. Todos os pais ou responsáveis foram informados sobre os objetivos e procedimentos do estudo e assinaram o Termo de Consentimento Livre e Esclarecido.

Foram avaliados 130 recém-nascidos pardos e brancos, de um a cinco dias de vida, do Setor de Alojamento Conjunto do Hospital Universitário da Universidade Federal de Mato Grosso do Sul, no período de junho a dezembro de 2016.

Foram incluídos nesse estudo recém-nascidos a termo, com APGAR maior ou igual a oito, em aleitamento materno exclusivo. Foram excluídos recém-nascidos da população indígena e quilombola; pré-termos; com complicações perinatais, condições clínicas instáveis, presença de anomalias craniofaciais, doenças neurológicas, síndromes genéticas visíveis no momento da avaliação, alimentação artificial, bem como, recém-nascidos de puérperas com soro positivo para o Vírus da Imunodeficiência Humana (VIH).

As avaliações foram realizadas após 24 horas do nascimento. A coleta de dados foi realizada por quatro avaliadoras, sendo a pesquisadora e três fonoaudiólogas da equipe do Hospital Universitário da Universidade Federal de Mato Grosso do Sul, devidamente treinadas e calibradas. Para essa fase, foi realizado um estudo piloto com a participação de 14 recém-nascidos. Ao final do treinamento, as avaliadoras atingiram grau de concordância acima de 90%. No estudo piloto foi utilizado o paquímetro de aço inoxidável temperado digital (marca Profield- Electronic Digital Caliper, 150 mm, resolução:0,01mm/0,0005”, precisão:+ou-0,02mm/+ou-0,001”, repetibilidade de 0,01mm/0,0005”, fabricado na Alemanha), a fita métrica e a régua maleável de 10 cm para a mensuração das medidas orofaciais.

A Figura 1 mostra os pontos que serviram como referência para as medidas antropométricas orofaciais: trichion (tr), ponto localizado na inserção do cabelo na linha mediana da testa; glabela (g), ponto mais proeminente na linha mediana entre as sobrancelhas; subnasal (sn), ponto situado medialmente no encontro da borda inferior do septo nasal com a superfície do lábio superior; labial superior (ls), localizado medialmente na linha do vermelhão do lábio superior; estômio (sto), ponto imaginário situado na região medial da intercessão entre a linha vertical mediana da face e a linha horizontal da rima da boca, quando os lábios estão fechados e os dentes ocluídos; gnátio (gn), ponto localizado na região mais inferior da borda inferior da mandíbula; canto externo do olho (ex), medialmente situado na borda externa do olho, tomando como referência o tecido duro; cheilion (ch), ponto localizado na comissura labial^(2,6,7,12)^.

**Figura 1:** Pontos antropométricos utilizados para as medidas **orofaciais** dos recém-nascidos

Legenda: tr = trichion; g = glabela; ex = canto externo do olho; sn = subnasal; ls = lábio superior; ch = cheilon; sto = estômio; gn = gnátio

Não foi observado diferença de valores entre o paquímetro, a fita métrica e a régua transparente, nos primeiros 14 recém-nascidos avaliados, assim foi escolhida a régua maleável transparente para avaliação, por ser considerada mais segura para recém-nascidos, uma vez que não é pontiaguda como o paquímetro. Pelo fato de a régua maleável ser transparente, foi possível assinalar com caneta hidrográfica o valor correspondente no momento da avaliação. Não foi realizada a análise estatística destas medidas no estudo piloto, apenas foram comparadas entre os avaliadores, com os diferentes instrumentos.

Após a verificação da exequibilidade da mensuração das medidas orofaciais pelo estudo piloto, foi realizada a avaliação dos 130 recém-nascidos. As medidas orofaciais dos recém-nascidos foram realizadas com uma régua maleável e transparente de 10 cm de comprimento, nos seguintes segmentos: alturas do terço superior (tr-g), do terço médio (g-sn) e do terço inferior da face (sn-gn); altura do filtro (sn-Is); e distância entre o canto do olho e a comissura labial do lado direito e esquerdo (ex-ch), conforme mostra a Figura 2. As medidas inicialmente foram tomadas em centímetros (cm), convertidas para mm e cada uma delas foi realizada três vezes, sendo realizado posteriormente o cálculo da média aritmética de cada medida. As mensurações foram realizadas com o bebê dormindo, deitado em berço hospitalar, na posição supina, com os lábios fechados, logo após a mamada. Os avaliadores utilizaram luvas durante toda avaliação e limparam a régua com álcool etílico hidratado 70º INPM, antes e após o procedimento.

**Figura 2:** Medidas antropométricas **orofaciais** dos recém-nascidos

Após as medições, foram coletadas, nos prontuários dos recém-nascidos, informações quanto ao sexo, peso e altura dos recém-nascidos. Para a comparação do peso e altura com as medidas antropométricas orofaciais foram utilizados os valores menor ou igual a mediana e maior que a mediana dessas variáveis.

Os dados obtidos foram tabulados e submetidos à análise estatística. A amostra foi caracterizada por meio de análise descritiva dos dados: média, desvio-padrão, coeficiente de variação (CV), valores mínimos (Min) e máximos (Max), com intervalo de confiança de 95%. Foi utilizado o programa IBM SPSS Statistics (*Statistical Package for the Social Sciences*), em sua versão 25.0, para a obtenção dos resultados, sendo aplicado o teste Mann-Whitney considerando o nível de significância de 5%.

## RESULTADOS

Dos 130 recém-nascidos avaliados, 69 (53%) eram do sexo feminino e 61 (47%) do sexo masculino. A média dos dias de vida dos recém-nascidos foi de 1,68 dias. A mediana do peso foi de 3,3 kg e da altura de 49 cm.

A Tabela 1 mostra os resultados obtidos por meio da comparação das medidas antropométricas orofaciais com o sexo dos recém-nascidos, mostrando que não foram encontradas diferenças significativas entre as medidas orofaciais dos recém-nascidos com o sexo.

**Tabela 1:** Comparação das medidas antropométricas **orofaciais** com o sexo

Variável	Sexo	n	Média	Desvio-padrão	Mínimo	Máximo	Percentil 25	Percentil 50 (Mediana)	Percentil 75	Valor de p
Terço superior da face **(mm**)	F	69	32,5	3,10	26,0	40,0	30,0	32,0	35,0	0,417
	M	61	32,7	3,00	25,0	40,0	30,0	33,0	35,0	
	Total	130	32,6	3,10	25,0	40,0	30,0	33,0	35,0	
Terço médio da face (**mm)**	F	69	29,6	2,30	25,0	38,0	28,0	30,0	30,0	0,883
	M	61	29,5	2,20	23,0	35,0	28,0	30,0	30,0	
	Total	130	29,6	2,30	23,0	38,0	28,0	30,0	30,0	
Terço inferior da face (**mm)**	F	69	29,3	3,00	22,0	40,0	27,0	30,0	30,0	0,070
	M	61	28,4	3,50	21,0	40,0	26,0	28,0	30,0	
	Total	130	28,9	3,20	21,0	40,0	27,0	29,0	30,0	
Canto externo do olho até comissura labial LE **(mm)**	F	69	39,4	2,30	33,0	44,0	38,0	40,0	40,0	0,814
	M	61	39,1	2,70	32,0	45,0	38,0	40,0	40,0	
	Total	130	39,3	2,50	32,0	45,0	38,0	40,0	40,0	
Canto externo do olho até comissura labial LD (**mm)**	F	69	38,7	2,80	25,0	44,0	38,0	39,0	40,0	0,651
	M	61	38,9	2,80	32,0	45,0	38,0	40,0	40,0	
	Total	130	38,8	2,70	25,0	45,0	38,0	39,5	40,0	
Filtro (**mm**)	F	69	8,70	1,30	5,00	11,0	8,00	9,00	10,0	0,944
	M	61	8,80	1,20	5,00	11,0	8,00	9,00	10,0	
	Total	130	8,70	1,30	5,00	11,0	8,00	9,00	10,0	

Teste Mann-Whitney

Legenda: F = Feminino; M = Maculino; LE = Lado Esquerdo; LD = Lado Direito

Quando comparadas as medidas antropométricas orofaciais com as medianas de peso, houve diferença significativa entre o peso e a distância entre o canto externo do olho e a comissura labial do lado direito e esquerdo (ex-ch), conforme descrito na Tabela 2.

**Tabela 2:** Comparação das medidas antropométricas **orofaciais** com o peso

Variável	Peso	n	Média	Desvio-padrão	Mínimo	Máximo	Percentil 25	Percentil 50 Valor/ Mediana	Percentil 75	Valor de p
Terço superior da face (**mm)**	≤ à mediana	65	32,6	3,10	25,0	40,0	30,0	33,0	35,0	0,911
	> à mediana	65	32,6	3,10	26,0	40,0	30,0	32,0	35,0	
	Total	130	32,6	3,10	25.0	40,0	30,0	33,0	35,0	
Terço médio da face **(mm)**	≤ à mediana	65	29,5	2,20	25,0	35,0	28,0	30,0	30,0	0,931
	> à mediana	65	29,6	2,30	23,0	38,0	28,0	30,0	30,0	
	Total	130	29,6	2,30	23,0	38,0	28,0	30,0	30,0	
Terço inferior da face (**mm**)	≤ à mediana	65	28,9	3,30	21,0	40,0	27,0	29,0	30,0	0,756
	> à mediana	65	28,8	3,20	22,0	40,0	26,5	29,0	30,0	
	Total	130	28,9	3,20	21,0	40,0	27,0	29,0	30,0	
**CEOCL** LE **(mm)**	≤l à mediana	65	38,7	2,70	32,0	45,0	37,0	39,0	40,0	0,003*
	**>** à mediana	65	39,9	2,00	35,0	44,0	39,0	40,0	41,0	
	Total	130	39,3	2,50	32,0	45,0	38,0	40,0	40,0	
**CEOCL** LD **(mm**)	≤l à mediana	65	38,3	3,10	25,0	45,0	37,0	39,0	40,0	0,016*
	**>** à mediana	65	39,2	2,20	33,0	44,0	38,0	40,0	40,0	
	Total	130	38,8	2,70	25,0	45,0	38,0	39,5	40,0	
Filtro **(mm)**	≤l à mediana	65	8,60	1,20	5,00	11,0	8,00	9,00	9,00	0,108
	> à mediana	65	8,90	1,30	5,00	11,0	8,00	9,00	10,0	
	Total	130	8,70	1,30	5,00	11,0	8,00	9,00	10,0	

Teste Mann-Whitney

* Significância Estatística

Legenda: CEOCL = Canto Externo do Olho até Comissura Labial; LE = Lado Esquerdo; LD = Lado Direito

Os resultados obtidos por meio da comparação entre as medidas antropométricas orofaciais e as medianas de altura estão demonstrados na Tabela 3, mostrando que não foram observadas diferenças significativas entre as medidas orofaciais dos recém-nascidos com a altura.

**Tabela 3:** Comparação das medidas antropométricas **orofaciais** com a altura

Variável	Altura	n	Média	Desvio-padrão	Mínimo	Máximo	Percentil 25	Percentil 50 (Mediana)	Percentil 75	Valor de p
Terço superior da face **(mm)**	≤l à mediana	66	32,4	2,90	25,0	40,0	30,0	33,0	35,0	0,820
	> à mediana	64	32,8	3,30	26,0	40,0	30,0	32,0	35,0	
	Total	130	32,6	3,10	25,0	40,0	30,0	32,0	35,0	
Terço médio da face **(mm**)	≤l à mediana	66	29,2	2,20	23,0	35,0	28,0	30,0	30,0	0,057
	> à mediana	64	29,9	2,30	25,0	38,0	28,0	30,0	31,0	
	Total	130	29,6	2,30	23,0	38,0	28,0	30,0	30,0	
Terço inferior da face **(mm)**	≤l à mediana	66	28,6	3,50	21,0	40,0	26,0	28,0	30,0	0,280
	> à mediana	64	29,1	2,90	24,0	40,0	27,0	29,0	30,8	
	Total	130	28,9	3,20	21,0	40,0	27,0	29,0	30,0	
**CEOCL** LE **(mm)**	≤l à mediana	66	39,0	2,70	32,0	45,0	38,0	40,0	40,0	0,095
	> à mediana	64	39,6	2,20	32,0	44,0	38,0	40,0	41,0	
	Total	130	39,3	2,50	32,0	45,0	38,0	40,0	40,0	
**CEOCL** LD (**mm**)	≤l à mediana	66	38,7	3,10	25,0	45,0	37,8	39,0	40,0	0,633
	> à mediana	64	38,9	2,30	33,0	44,0	38,0	40,0	40,0	
	Total	130	38,8	2,70	25,0	45,0	38,0	39,5	40,0	
Filtro (**mm**)	≤l à mediana	66	8,60	1,30	5,00	10,0	8,00	9,00	9,30	0,438
	> à mediana	64	8,80	1,30	5,00	11,0	8,00	9,00	10,0	
	Total	130	8,70	1,30	5,00	11,0	8,00	9,00	10,0	

Teste Mann-Whitney

Legenda: **CEOCL =** Canto Externo do Olho até Comissura Labial; LE = Lado Esquerdo; LD = Lado Direito

## DISCUSSÃO

O presente estudo foi motivado devido à escassez de dados a respeito das medidas antropométricas orofaciais de recém-nascidos, sendo pioneiro na investigação dessas medidas na região Centro-Oeste do Brasil.

A média do peso dos recém-nascidos avaliados foi de 3.302 g e a média da altura de 49 cm, sendo esses valores próximos dos encontrados por Oliveira et al.^(19)^ em 450 recém-nascidos avaliados, cuja média de peso foi de 3.278 g e de altura de 48,8 cm.

Quando comparadas as medidas antropométricas orofaciais com sexo, não houve diferença estatística, mostrando que o sexo parece não interferir nessas variáveis. Esses achados diferem dos resultados encontrados por Medeiros et al.^(18)^, que relataram existir diferenças das medidas orofaciais entre os sexos logo ao nascimento, sempre maiores no sexo masculino. Em outros estudos foram encontradas diferenças significativas entre altura do filtro e sexo^(20,21)^, distância das comissuras da boca entre os sexos^(4)^.

Foram identificadas divergências nos resultados destas pesquisas, isto pode ser explicado pelo fato que estes estudos utilizaram distintas metodologias e foram realizados em diferentes regiões de vários países. A literatura refere que as dimensões da cabeça e da face variam de acordo com a raça e a zona geográfica^(4)^.

Este estudo também evidenciou diferença estatisticamente significante quando comparadas as medidas antropométricas orofaciais da distância entre o canto do olho e a comissura labial direita e esquerda com o peso ao nascimento. Assim, pesos maiores permitem observar valores efetivamente maiores da distância entre o canto do olho e a comissura labial esquerda e direita; e pesos menores, valores menores. A escassez de estudos não permite comparar os dados encontrados, porém, é possível inferir que as medidas da distância entre o canto do olho e a comissura labial são influenciadas pelo peso. Uma possível explicação para esse achado pode estar no fato de, talvez, em bebês com peso maior as bolsas de gorduras serem mais volumosas, e como a régua maleável se molda à estrutura, aumentaria a medida da distância entre o canto do olho e a comissura labial.

Ao comparar a altura com as medidas antropométricas orofaciais não foram encontradas diferenças estatisticamente significantes. A literatura escassa nessa área e para essa população dificultou a comparação dos achados com outros estudos. Este fato pode estar relacionado devido ao crescimento facial ser mais acentuado nos primeiros dois anos de vida, através da amamentação e não necessariamente ao nascimento.

Como limitação da presente pesquisa, podemos citar a falta de pareamento dos grupos quanto ao sexo, havendo uma predominância do sexo feminino. Considerando a miscigenação étnica brasileira, os valores encontrados no Estado de Mato Grosso do Sul não podem ser generalizados para recém-nascidos de outras regiões do país. Assim, futuros estudos multicêntricos, utilizando a mesma metodologia do estudo proposto, com uma amostra maior, contribuirão para o conhecimento do perfil das medidas orofaciais dos recém-nascidos brasileiros.

## CONCLUSÃO

Não houve diferença nas medidas antropométricas orofaciais de recém-nascidos a termo quando comparadas com sexo e altura; porém, quando comparadas com peso, há diferença nas medidas da distância entre o canto do olho e a comissura labial do lado direito e esquerdo.
